# PMRD: a curated database for genes and mutants involved in plant male reproduction

**DOI:** 10.1186/1471-2229-12-215

**Published:** 2012-11-15

**Authors:** Xiao Cui, Qiudao Wang, Wenzhe Yin, Huayong Xu, Zoe A Wilson, Chaochun Wei, Shenyuan Pan, Dabing Zhang

**Affiliations:** 1 State Key Laboratory of Hybrid Rice, School of Life Sciences and Biotechnology, Shanghai Jiao Tong University, Shanghai, 200240, China; 2 School of Life Sciences, Jiangsu Normal University, Xuzhou, 221116, China; 3 School of Biosciences, University of Nottingham, Loughborough, Leicestershire, Nottingham, LE12 5RD, UK; 4 Department of Bioinformatics and Biostatistics, School of Life Sciences and Biotechnology, Shanghai Jiao Tong University, Shanghai, 200240, China

**Keywords:** Plant male reproduction, Database, Gene, Mutant, Pollen, Anther

## Abstract

**Background:**

Male reproduction is an essential biological event in the plant life cycle separating the diploid sporophyte and haploid gametophyte generations, which involves expression of approximately 20,000 genes. The control of male reproduction is also of economic importance for plant breeding and hybrid seed production. With the advent of forward and reverse genetics and genomic technologies, a large number of male reproduction-related genes have been identified. Thus it is extremely challenging for individual researchers to systematically collect, and continually update, all the available information on genes and mutants related to plant male reproduction. The aim of this study is to manually curate such gene and mutant information and provide a web-accessible resource to facilitate the effective study of plant male reproduction.

**Description:**

Plant Male Reproduction Database (PMRD) is a comprehensive resource for browsing and retrieving knowledge on genes and mutants related to plant male reproduction. It is based upon literature and biological databases and includes 506 male sterile genes and 484 mutants with defects of male reproduction from a variety of plant species. Based on Gene Ontology (GO) annotations and literature, information relating to a further 3697 male reproduction related genes were systematically collected and included, and using in text curation, gene expression and phenotypic information were captured from the literature. PMRD provides a web interface which allows users to easily access the curated annotations and genomic information, including full names, symbols, locations, sequences, expression patterns, functions of genes, mutant phenotypes, male sterile categories, and corresponding publications. PMRD also provides mini tools to search and browse expression patterns of genes in microarray datasets, run BLAST searches, convert gene ID and generate gene networks. In addition, a Mediawiki engine and a forum have been integrated within the database, allowing users to share their knowledge, make comments and discuss topics.

**Conclusion:**

PMRD provides an integrated link between genetic studies and the rapidly growing genomic information. As such this database provides a global view of plant male reproduction and thus aids advances in this important area.

## Background

Male reproduction is a complex and highly coordinated biological process that includes the development of the male reproductive organ, the stamen, that contain the microspores/pollen, as well as subsequent pollen release, pollination, pollen tube growth, guidance, reception, gamete migration and finally fertilization [1-6]. The stamen comprises an anther with multiple specialized cells/tissues for the production of viable pollen and a filament that supports the anther. Microspore/pollen development requires meiotic and subsequent mitotic divisions, and numerous cooperative functional interactions between the gametophytic and sporophytic tissues within the anther. Pollen development needs precise spatiotemporal expression of genes, orchestrated activity and localized control of enzymes, cell-to-cell communication, cell development and differentiation [2,6]. Furthermore, disruption of gene expression by environmental effects, or genetic mutations, frequently results in reduced fertility, or complete male sterility, causing loss of agricultural yield. Control of plant fertility is also of economic importance with some male sterile lines used in agriculture for crop improvement, for example in breeding of super hybrid rice [7].

Due to the importance of male reproduction, much effort has been applied to understand the molecular regulation of plant male reproduction. Transcriptome analysis has indicated that more than 20,000 genes are expressed in rice (*Oryza sativa*) developing anthers and about 18,000 in Arabidopsis (*Arabidopsis thaliana*) pollen [8-10]; suggesting extensive gene expression changes during anther development and pollen formation [11]. Furthermore, recent forward and reverse genetic studies have identified a large number of male sterile mutants and related genes [1,12,13]. However, it is time-consuming and inefficient for individual researchers to access accurate information on male reproduction in plants. This is particularly relevant in the context of comparative analysis between species.

To bridge the gap between genetic studies and genomic information in plant male reproduction, we systematically collected male sterile mutant and gene information by manual curation, and created the PMRD (Plant Male Reproduction Database) database. This database provides a bi-directional integration of the rapidly growing genomic data and knowledge from genetic studies, which will undoubtedly improve our understanding of the mechanisms of plant male reproduction. PMRD functions not only as a high quality curated database for browsing and retrieving knowledge on genes and mutants in plant male reproduction, but also as a dynamic website with build-in bioinformatics tools to access genomic information. Moreover, PMRD is designed with knowledge sharing features that include wiki and forum tools to facilitate community annotation, information sharing and education.

## Construction and content

### Collection of plant male reproduction related genes

Genes included in PMRD are divided into two categories: male sterile genes (MS genes) and male reproduction related genes (MR genes). The differences between MS genes and MR genes is that the function of MS genes has been demonstrated by analyzing the mutants showing reduced male fertility or transmission efficiency, whereas, MR genes mean that the MR genes have the putative function in male reproduction without genetic evidence. MS genes were identified from literature and biological database searches. MR genes were identified based upon GO annotations, the phenotypes of TAIR germplasms and expression information in literature [14]. In order to establish a repository of literature for manual curation, we extensively collected publications on genetic and molecular studies of plant male reproduction through Pubmed and journal specific database searches. A total of 370 full-text publications were retrieved, including 143 papers for rice (*Oryza sativa*), 187 papers for *Arabidopsis thaliana* and 40 papers for a further 31 plant species. From this local repository of literature 343 MS genes and 321 MS mutants were identified. Next, we collected 163 MS mutants from two rice databases: Oryzabase and China Rice Data Center [15,16]. To identify MR genes, we collected 41 GO terms associated with plant male reproduction from the GO Consortium [14]. Subsequently we mapped the 41 GO terms onto annotations from the RAP-DB, TAIR and PLAZA websites [17-19] (See Additional file 1). Regarding MR genes identified in the literature, we collected 3697 MR genes. Therefore when combined with the MS genes, we have identified 4203 genes and 484 mutants in 33 species that are implicated as involved in plant male reproduction (Table 1).

**Table 1:** PMRD current data status

	***O. sativa***	***A. thaliana***	**Other (31) species**	**Total count**
MS genes	227	227	52	506
MR genes	119	321	3257	3697
MS mutants	243	241	-	484
References	143	187	40	370

MS genes: male sterile genes; MR genes: male reproduction related genes.

### Data entry and curation

Curation of information from publications into a well-structured searchable repository of knowledge is a critical step in biological database construction. This included manual review of papers, identification of biological entities, definition of the experimental methods used, conversion of experimental results and phenotypic observations into a standard format, and summarizing gene function data. In the PMRD curation process, papers were initially examined and checked whether appropriate for inclusion as an MS/MR gene in PMRD. The criterion for inclusion as an MS gene was that mutation of the gene must cause defects in male reproduction. Once identified the full-name, gene symbol and a brief description of the gene were obtained. Information was collated associated with the gene product expression pattern, molecular and biological function. Genes in rice and Arabidopsis were then mapped onto RAP-DB and TAIR locus, and included in PMRD. For other species, gene names mentioned in the papers were used. Gene expression assays in both rice and Arabidopsis were curated in detail using controlled anatomy and stage vocabularies. If the papers included genetic or transgenic studies of mutants, the curators captured the following information: mutant names, mutated genes, mutagenesis methods, dominance, mutant phenotypes and male sterility categories. All curated information was checked and confirmed by senior experts in this field.

### Database design implementation

**Figure 1:**
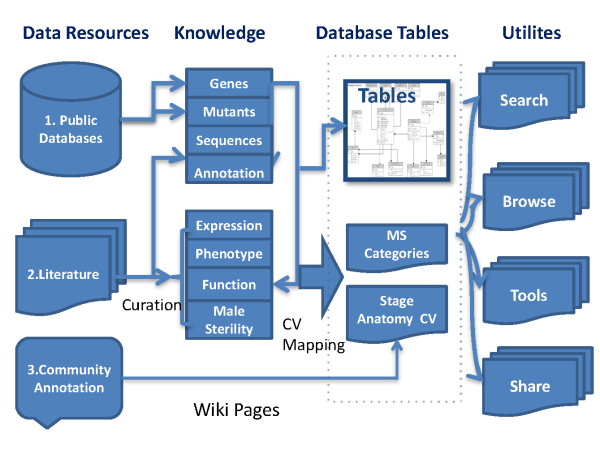
**Overview of the PMRD database architecture.** Plant Male Reproduction Database (PMRD) is a comprehensive resource for browsing and retrieving knowledge about genes and mutants related to plant male reproduction. PMRD brings together three main sources of knowledge: (1) general genomic information from public databases; (2) detailed curation of genetic studies from the literature; (3) public annotation from the research community. Curated information and genomic information are stored in relational database tables that are accessed by a number of online utilities.

PMRD functions as a database system that brings together three main sources of knowledge: 1) general genomic information from public databases; 2) detailed curation of genetic studies from the literature; 3) public annotation from the research community (Figure 1). In the genomic annotation section, the chromosomal location, sequence, GO terms, KEGG pathway information and Interpro annotations are displayed [14,15,17,18,20,21]. Plant male reproduction-associated microarray datasets were downloaded from GEO [22]. To provide detailed anatomical information on mutant phenotype and gene expression we firstly designed tags and controlled vocabulary (CV), which were then used to normalize the information during the curation process. Controlled vocabulary for the development stages and anatomy was set according to publically accepted standards [1,23]. The curated information in PMRD includes: summaries of genes function, gene expression patterns, mutant background, mutagenesis methods, descriptions of mutant phenotypes and male sterile type definitions. Genes for anther development and pollen formation were collated and the information organized in a two-dimensional module displayed on a webpage, which associates genes and mutants with stages and tissues, allowing multiple ways to browse genes and mutants of interest. For other male reproduction processes we capitalized upon community annotation, and created 4 online data collection tables, including “Pollination”, “Pollen Germination and Tube Growth”, “Guidance and Perception”, “Migration and Fusion”. We also integrated Mediawiki engine into PMRD, thus allowing users to contribute their knowledge on mutants, development stages, anatomy, and to create other topics that they have interests in. Finally, a forum was also setup to facilitate discussions.

PMRD runs on an Nginx server using MySQL as the storage engine. The web interface is implemented using PHP and JavaScript libraries [24]. Mediawiki engine was integrated into PMRD as a community annotation tool [25]. Different interfaces in PRMD were wrapped by Joomla content management system for site maintenance [26]. The web page works well in all major browsers.

### Utility and discussion

#### Database web interface

**Figure 2:**
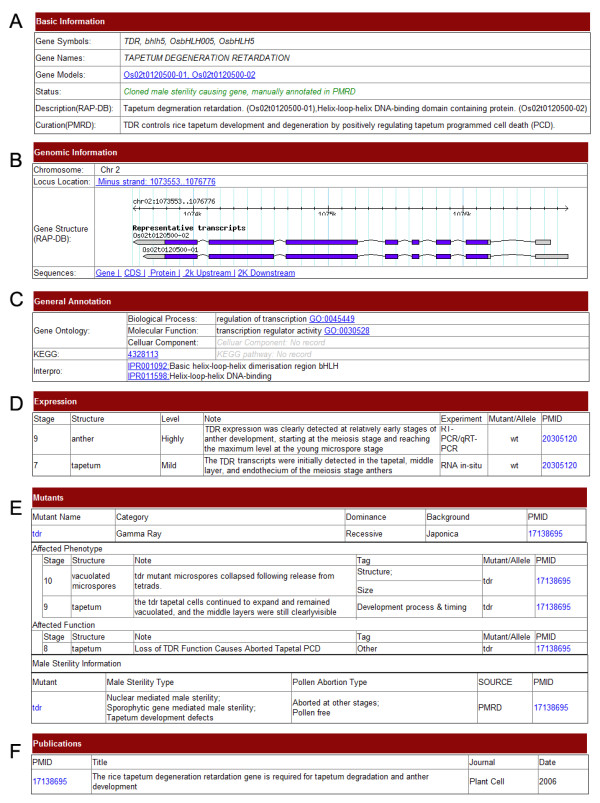
**Screenshot of gene information.** The detailed information page of an MS gene consists of six sections. (**A**) Basic Information, (**B**) Genomic Information (**C**) General Annotation, (**D**) Expression, (**E**) Mutant, containing related mutant information, (**F**) Publications.

**Figure 3:**
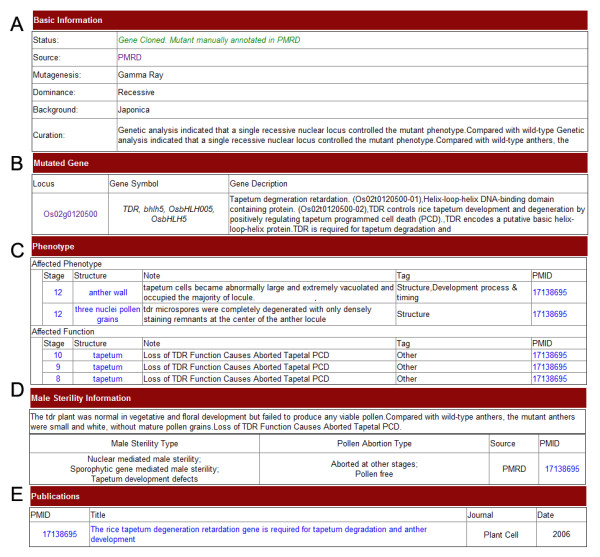
**Screenshot of mutant information.** The detailed information page of an MS mutant consists of five sections. (**A**) Basic Information, (**B**) Mutated gene information (**C**) Phenotype, (**D**) Male sterility information, (**E**) Publications.

We developed a user-friendly web interface for searching and browsing information in PMRD. Users can easily search genes by names, identifiers, sequences, expression, phenotypes and male sterile categories of relative mutants. Since the data structure for different species is not the same, web pages for searching and browsing are grouped into rice, Arabidopsis and other species in the main PMRD website menu. To make information retrieval convenient and precise, search pages are designed to include both simple and advanced options. The web page displaying information on MS genes contains six sections (Figure 2). The first section displays “Basic Information” of the gene, including gene symbols, gene names, description of genes from external databases and function as curation by PMRD staff. The second section contains “Genomic Information”, such as locations, gene structures and sequences. The third section displays “General Annotation” retrieved from external databases, including GO terms, KEGG pathway information and Interpro protein signatures [14,20,21]. The forth section displays the “Expression Pattern” of the gene. Expression information for rice and the Arabidopsis were obtained from literature curation and TAIR annotation [18]. The fifth part summarizes “Mutant” information of the gene, including mutants, phenotypes, and male sterile categories. Male sterile categories indicate the pollen abortion type, which were set according to plant ontology (PO) and rice knowledge bank [27,28]. A mutant can be assigned to more than one category. If detailed male sterile information of the mutant could not be obtained from data sources, it was assigned as “not defined”. The sixth section shows the “Publications” related to the genes. Web pages displaying mutant information are organized into five sections (Figure 3). Basic information includes mutagenesis method, dominance, background and a short description of the mutant. The following section includes information and links for the mutated genes. The third section displays curation of mutant phenotype observations. The last two sections display male sterility information and related publications. In case of a very long page, the user can collapse/expand the panel for each section, however because of heterogeneous data sources, not all contain complete datasets for all of the sections mentioned above.

**Figure 4:**
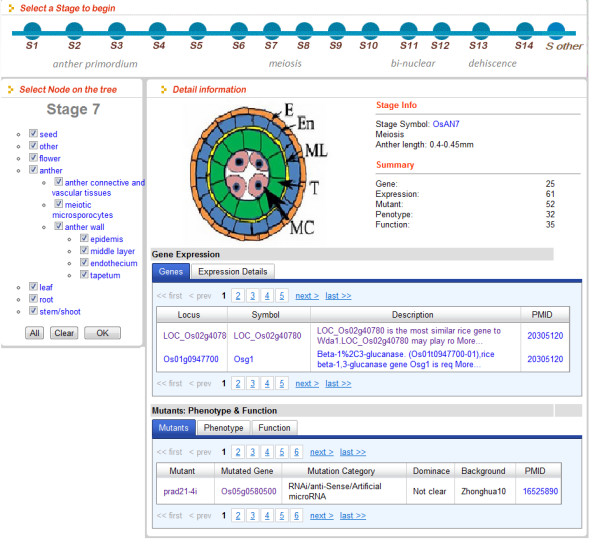
**Screenshot of the “Browse Anther Development” page for rice and Arabidopsis.** The browse anther development page was created as a hub to integrate gene, mutant, expression and phenotype information and arrange it in a two-dimensional way according to stage and tissue information.

**Figure 5:**
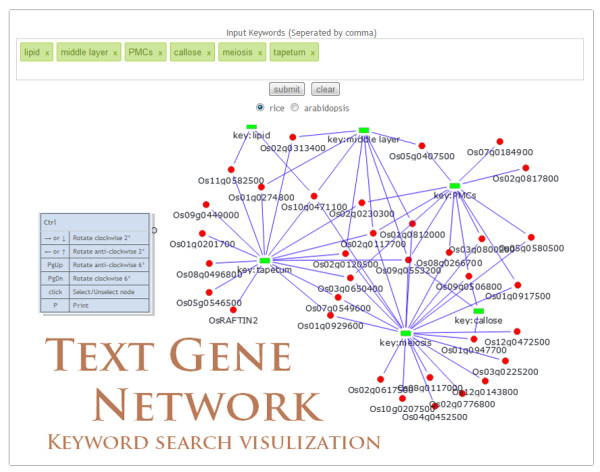
**Text-Gene Network.** A tool to display keyword search results in an intuitive and informative way. Connections are drawn between MS genes and keywords if the keywords appeared in the data entries related to the genes, including gene description, expression, related mutant phenotypes and Gene Ontology annotations.

**Figure 6:**
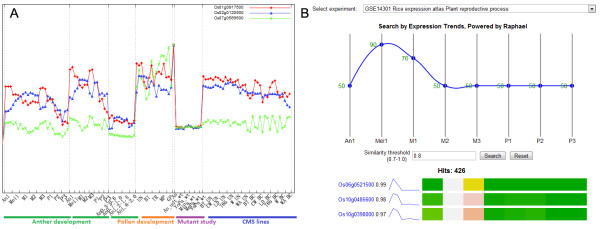
**Microarray data visualization and search interface for rice and Arabidopsis.** (**A**) Visualization of gene expression pattern from multiple microarray datasets related to plant male reproduction, (**B**) Searching gene expression patterns in a specific microarray experiment.

PMRD also provides a variety of tools for information retrieval and display. “Browse page” was created (Figure 4) as a hub page to integrate information on genes, mutants, expression and phenotypes into a single interface according to stages and tissues during different male reproduction processes. The Ajax technique was employed to navigate through stages and tissues without refreshing. To enable more intuitive and informative multiple keywords searches, we developed a tool to visualize keywords-gene relationship using CanvasXpress and CytoscapeWeb (Figure 5) [24,29]. This draws a connection between an MS gene and a keyword if the keyword appeared in the data entries related to the gene, including gene description, expression, related mutant phenotypes and GO annotations. For microarray data, the user can browse and search expression information on the microarray visualization page and microarray search page (Figure 6). We also provide a tool for BLAST searching for rice and Arabidopsis to help users search genes by sequences and ID converter for different databases [18,30-33]. Finally, a wiki page with an easy to use editor plug-in has been setup to promote community information contributions; we encourage the users to contribute their knowledge in the wiki page and recommend literatures to us.

### Future directions

Comparative functional genomics study is an emerging approach that relies upon the application of the vast accumulated knowledge available for model species to less characterized species. Recently, a number of comparative, or functional genomics websites for plants have been developed, such as PLAZA, Phytozome, the Floral Genome Project, MoccaDB, SolRgene and BRAD [19,34-38]. As more plant genome sequences become available, it will be interesting to extend and apply the current knowledge in PMRD for comparative studies. Future versions of PMRD will provide cross-species tools for comparing and mining male reproduction related genes. Finally there is an urgent need for automatic literature curation, since manual text curation is a challenging job for annotators, which requires much expertise and devotion. A number of gateway databases for model species have adopted text-mining tools. The Mouse Genome Informatics has initiated a dictionary based text mining tool to help biocuration [39]. Flybase has developed natural language processing and automatic experimental information categorization tools to aid curation [40,41]. At the moment the data sources of PMRD are mostly literature from genetic and molecular studies. In such papers, information is often organized into discernable sections, such as initial characterization of a gene, gene expression assays, and morphological phenotype observations, etc. Two text-mining tools are currently available for the Arabidopsis [42,43]; it is hoped that such text-processing software will be used in future updates and maintenance of the database.

Finally, plant male reproduction covers a wide range of biological processes and the improvement of PMRD requires continuous effort and community contributions. The first version of PMRD is based on data collected mainly from anther and pollen development. For future updates, we have opened online data collection tables to extend the detailed coverage of related topics.

## Conclusions

Plant Male Reproduction Database (PMRD) is a comprehensive resource for browsing and retrieving knowledge about genes and mutants related to plant male reproduction. Currently, PMRD holds information for 4203 genes and 484 mutants associated with plant male reproduction across 33 plant species. The two major model plant species, rice and Arabidopsis, have the greatest number of entries and most detailed curation. The ultimate goal of the database is to extend this further to provide a dynamic and comprehensive information resource with associated data mining tools to aid research in plant male reproduction.

### Availability and requirement

The PMRD database is freely accessible at [44].

## Abbreviations

CV: Controlled vocabulary; GEO: Gene Expression Omnibus; GO: Gene ontology; KEGG: Kyoto Encyclopedia of Genes and Genomes; MR gene: Male reproduction related gene; MS gene: Male sterile gene; PO: Plant ontology; RAP-DB: The Rice Annotation Project Database; TAIR: The Arabidopsis Information Resource.

## Competing interests

The authors declare that they have no competing interests.

## Authors’ contributions

XC, DW and HY designed and implemented the database, website pages and on-line tools. XC, DW, ZAW, DZ, WY are responsible for data collection, manually curation and quality control. PS, CW participated in design of the database schema. ZAW and DZ conceived the study. XC, ZAW, DZ drafted the manuscript. All authors read and approved the manuscript.

## Supplementary Material

Additional file 1 Gene ontology terms used to identify male reproduction related genes. Click here for file
